# CAUTI’s next top model – Model dependent *Klebsiella* biofilm inhibition by bacteriophages and antimicrobials

**DOI:** 10.1016/j.bioflm.2020.100038

**Published:** 2020-11-11

**Authors:** Eleanor M. Townsend, John Moat, Eleanor Jameson

**Affiliations:** School of Life Sciences, Gibbet Hill Campus, The University of Warwick, Coventry CV4 7AL, United Kingdom

**Keywords:** Phage therapy, Clinical biofilms, Urinary-catheter model, In vitro models

## Abstract

*Klebsiella* infections, including catheter associated urinary tract infections, are a considerable burden on health care systems. This is due to their difficulty to treat, caused by antimicrobial resistance and their ability to form biofilms. In this study, we investigated the use of a *Klebsiella* phage cocktail to reduce biofilm viability. We used two methodologies to investigate this, a standard 96-well plate assay and a more complicated Foley catheter-based model. The phage cocktail was used alone and in combination with clinically relevant antibiotic treatments. Viability was measured by both a resazurin based stain and colony forming unit counts, of cells sloughed off from the biofilm. We showed that phage infection dynamics and host survival vary significantly in different standard laboratory media, presumably due to the expression of different surface receptors and capsule composition by the bacteria effecting phage binding. This underscores the importance of a realistic model for developing phage therapy.

We demonstrate that bacteriophage-based treatments are a viable option for preventing *Klebsiella* colonisation and biofilm formation on urinary catheters. Phage cocktails were able to significantly reduce the amount of biofilm that formed when they were present during early biofilm formation. The phages used in this study were unable to significantly reduce a pre-formed mature biofilm, despite encoding depolymerases. Phages applied together with antimicrobial treatments, showed synergistic interactions, in some cases the combined treatment was much more effective than antimicrobial treatments alone.

We show that phage cocktails have the potential to prevent *Klebsiella* biofilms in catheters, if used early or as a preventative treatment and will work well alongside standard antibiotics in the treatment of catheter-associated urinary tract infections (CAUTI).

## Introduction

Carbapenem-resistant and third-generation cephalosporin-resistant Enterobacteriaceae, such as *Klebsiella* sp. have been named by the World Health Organisation (WHO) as one of the critical priority bacteria in the fight against antibiotic resistance [1]. *Klebsiella* species cause a variety of opportunistic infections including urinary tract infection (UTI), pneumonia, septicaemia, wound infection, and infections in vulnerable patients including neonates and intensive care patients [2]. *Klebsiella* species can encode virulence factors which make them efficient pathogens, but of most concern are the high levels of multiple antibiotic resistance mechanisms found within the genus. Resistance rates within pathogenic *Klebsiella* sp. have increased exponentially to most available antimicrobial drugs, with multidrug resistance seen in UTI associated *Klebsiella* [3,4] and cases of pan-resistant *Klebsiella* described [5,6]. Antibiotic resistance within the *Klebsiella* genus is mediated by antibiotic resistance genes encoded for in both their chromosome and mobile plasmids [[7], [8], [9]]. This increased resistance is often associated with an increased risk of mortality [10].

Complicating the antibiotic resistance of *Klebsiella* species is their ability to form biofilms. It has been shown with some antibiotics, that lack of penetration into the biofilm prevents killing of *Klebsiella*, however there are other mechanisms at work [11]. The thickness of mature *Klebsiella* biofilm has been implicated in increased antibiotic tolerance as well as biofilm heterogeneity [12]. Nutrient limitation, leading to lack of growth, has also been shown to contribute to antibiotic tolerance even with full penetration of the drug [12,13]. It has also been shown that sub-lethal concentrations of antibiotics, often found within biofilms, can increase the virulence, biofilm formation and antibiotic resistance of *Klebsiella* species [14,15]. These biofilm properties confer the benefit of antibiotic tolerance to the organisms that form them whilst growing in this phenotype, which differs from antibiotic resistance conferred by antibiotic resistance genes. However, *Klebsiella* strains that possess the ability to form rigid biofilms are also more likely to be Extended-Spectrum Beta-lactamase (ESBL) producers [16], as well as having increased ability to transfer plasmids within biofilm [15], which further complicates therapy.

The ability to form biofilms means *Klebsiella* spp. are able to colonise medical devices, leading to central line associated sepsis, ventilator associated pneumonia, and catheter associated urinary tract infections. *Klebsiella penumoniae* and *Klebsiella oxytoca* have both been found to be common causes of Catheter Associated Urinary Tract Infections (CAUTI) with high rates of antimicrobial resistance [[17], [18], [19]].

Bacteriophages offer an alternative method of treating biofilm infections. The lytic lifecycle of phages is a virulent lifestyle that results in bacterial death, this lifestyle can be taken advantage of for therapeutic use. Lytic phages are preferential for therapeutic use, because temperate phages may increase the fitness of their hosts, as well as the potential presence of antimicrobial resistance and toxin genes in temperate phages [20]. Utilising lytic phages as antibacterial agents was first described by d’Hérelle in 1919, and he summarised his successes in a review in 1931 [21]). Phages offer a number of advantages over antibiotics as anti-biofilm agents, including increased specificity, accuracy and potency [22,23]. Phages are able to degrade the biofilm extracellular matrix, allowing antimicrobial penetration, lysis of bacterial cells and extensive biofilm disruption [24,25].

Phage coating for catheters have already been developed for other common CAUTI pathogens such as *Proteus mirabilis*, *Escherichia coli*, *Pseudomonas aeruginosa* [26,27], and combinations of these bacteria in polymicrobial biofilms [28,29]. Small scale clinical trials have been conducted, which show the efficacy of phages on catheters *in vivo* [30]. However, to our knowledge, there currently exists no equivalent treatment for *Klebsiella* caused CAUTI.

CAUTI associated *Klebsiella* present a novel bacteriophage target and unique challenges [31]. demonstrated that their UTI Klebsiella isolates showed a mucoid phenotype and that higher drug-resistant corelated to biofilm formation and polysaccharide production in *Klebsiella*. Polysaccharide production is widespread in *Klebsiella* sp., with 77 different capsule types having previously been serologically defined [32,33]. These protective polysaccharide capsules endow resistance to not only antibiotics, but also protists and phages [34], [65]. In response phage tail fibres are highly diverse and frequently rearranged, often consisting of enzymes that can degrade bacterial structures, such as depolymerases that target bacterial capsules [35]. These enzymes may facilitate diffusion of phage, or antimicrobial drugs, into the biofilm, which is a commonly cited cause for antimicrobial tolerance in biofilms [12].

In this paper, we investigate the potential for a number of previously described *Klebsiella* phages [36] to be used as anti-biofilm agents in preventing CAUTI. Using a number of urinary-isolated *Klebsiella* species we developed an *in vitro* model of CAUTI biofilms, which we used to test combinations of antimicrobial drugs alongside a phage cocktail. Our *in vitro* model was based around the use of artificial urine media (AUM) and sections of Foley catheter. We showed that the phage cocktail and antimicrobial therapy are in some cases able to complement each other’s activity, leading to an enhanced anti-biofilm effect.

## Materials and methods

### Culture conditions and standardisation

Strains used in this study were from culture collections or clinical strains. The clinical strains (*Klebsiella pneumoniae* 170723, *Klebsiella oxytoca* 170748, *Klebsiella pneumoniae* 170958, and *Klebsiella oxytoca* 171266) were all isolated in the UK from urinary tract infections, with or without the presence of a medical device, described in Table 1. Type strain *Klebsiella pneumoniae* subsp. *pneumoniae* 30104 was obtained from the DSMZ culture collection (Leibniz Institute, Germany). All isolates were stored long-term at −80 ​°C and short-term at 4 ​°C. Strains were maintained on LB agar and propagated in Cation-adjusted Mueller Hinton Broth (CAMHB, Sigma-Aldrich, Gillingham, UK). After growing to exponential phase (OD_600nm_ 0.4–0.6), cells were washed with phosphate buffered saline (PBS, Oxoid, Basingstoke, UK; 0.137 ​M NaCl_2_, 3 ​mM KCl, 8 ​mM Na_2_HPO_4_, 1.5 ​mM KH_2_PO, pH 7.3) and normalised based on optical density at 600 ​nm before experimental work.

**Table 1:** Description of *Klebsiella* clinical isolates used in this work. *Klebsiella* clinical isolates used in this work are described within this table including their Genus, species, strain identification (ID) number and source of isolation.

**Species**	**Strain ID**	**Source**
*Klebsilla pneumoniae*	170723	Urine
*Klebsiella oxytoca*	170748	Catheter Specimen Urine
*Klebsiella pneumoniae*	170958	Urine
*Klebsiella oxytoca*	171266	Urostomy urine

### Bacterial growth media

The bacterial growth media used within this study is specified for each technique, includes Cation-adjusted Mueller Hinton Broth (CAMHB, Sigma-Aldrich, Gillingham, UK), Luria Broth (LB, Sigma-Aldrich, Gillingham, UK), M9 minimal media [37] supplemented with 5 ​mM glycerol and Artificial Urine Medium (AUM) [38].

### Bacteriophages

Bacteriophages used in this paper were isolated and characterised by our lab group; the bacteriophages are genetically distinct, span four different phage genera and demonstrated different host ranges described in Ref. [36]. The phage used in this study, their isolation host strain, and source have been described in Table 2. Single phages were used for treatment, as well as a cocktail consisting of all six phages, in equal portions, as determined by plaque forming units (PFU ml^−1^). Phages are used as a cocktail for therapeutic use, with the aim of overlapping host range and activity to overcome any specificity the individual phage may have. Strain susceptibility to each phage is described in Table S1.

**Table 2:** Description of phage used in this work. Phage used in this work are described within this table including their full name, isolation host and source of isolation.

**Full Name**	**Isolation host**	**Source**
vB_KppS-Samwise	*Klebsiella pneumoniae* subsp *pneumoniae* 30104	Slurry
vB_KppS-Jiji		Pond water
vB_KppS-Strom		Sewage - storm tank storage
vB_KppS-Pokey		Sewage - anoxic sludge
vB_KppS-Anoxic		Sewage - anoxic sludge
vB_KoM_Flushed	*Klebsiella pneumoniae*170958	Sewage - mixed liquor

### Biofilm formation in cell-culture plates

All *Klebsiella* strains were standardised to 1 ​× ​10^2^ ​CFU/mL in CAMHB. CAMHB was selected because it is the industry standard method to measure the efficacy of antimicrobials using the minimum inhibitory concentration (MIC) method. Into a 96-well flat bottom cell culture treated plate (CytoOne Plate, Starlab, UK), 200 ​μL of cell suspension was added. Culture plate biofilms were used as a simple, *in vitro* biofilm model to quickly determine the ability of *Klebsiella* strains to form biofilms and bacteriophages ability to inhibit biofilm formation and biofilms. To form a biofilm, the plates were incubated statically for 16 ​h at 37 ​°C. After 16 ​h, biofilm viability had stabilised and therefore were now considered mature (Fig. S1). All procedures were carried out in a Class II microbiological safety cabinet. Negative controls containing no inoculum, and positive controls which had no treatment applied, were included in each plate. All testing was carried out in triplicate, on three separate occasions.

Phage and Antimicrobial Treatment in cell-culture plates.

For investigating phage inhibition of biofilm formation, 10 ​μL of phage suspension was added to the well before any bacteria were added. Biofilms were then allowed to form, as described above.

To treat mature biofilms, the biofilms were first washed with PBS to remove any planktonic cells. Following this, 10 ​μL of phage stock suspension (1 ​× ​10^2^ ​PFU/mL) was added to each well. Meropenem was selected for treatment because the *Klebsiella* strains used showed varying degrees of susceptibility based on the MIC’s calculated for each strain (Table S2). All strains had reduced susceptibility in the biofilm phenotype. For treatment with meropenem for 5 ​h, the drug was suspended in CAMHB at 128 ​mg/L and 64 ​mg/L for high and low concentrations respectively. Untreated controls and negative controls were included in every experiment. The experiments were performed in triplicate.

### Phage infection in different media

*Klebsiella* strains were grown to exponential phase in four different media (LB, CAMHB, M9 supplemented with 5 ​mM glycerol and AUM [38]). The strains were then diluted to an OD_600nm_ which was equivalent to approximately 1 ​× ​10^7^ ​CFU/mL and 150 ​μL of bacterial suspension was added per well in a 96-well microtitre plate. The wells then had either 50 ​μL phage cocktail (test wells) or 50 ​μL media (control wells) added.

The 96-well microtitre plate was sealed and incubated at 37 ​°C with shaking at 200 ​rpm in between readings and 500 ​rpm for 30 ​s before readings in a FLUOstar® Omega plate reader (BMG Labtech, Aylesbury, UK). The optical density of the cultures was recorded over 24 ​h, with readings at OD_600nm_ taken every 5 ​min. Each plate contained technical duplicates for each *Klebsiella* strain in each media and was performed in biological triplicate.

The area under the curve (AUC) was calculated using MatLab, following the methodology of [39]. The AUC method allowed direct comparison of the phages in each host and media in a high-throughput and reproducible manner. The ratio of bacteria-only control AUC to phage infected AUC was calculated for each growth media. For each strain, the AUC ratio was normalised by Ln transformation and then each media was compared using a two-tailed t-test to identify where phage infection was affected by the growth media.

Biofilm formation and treatment in simple catheter model.

Foley catheters (Folatex ref. AA1B16 Ch/Fr 16/5.33, 30–45 mL/cc, silicone coated latex urinary catheter/straight/2-way) were cut into 1.5 ​cm long sections, aseptically. A bacterial suspension of 1 ​× ​10^2^ ​CFU/mL in CAMHB was formed by diluting from an exponentially growing culture. The catheter sections were incubated in the bacterial suspension for 2 ​h, at 37 ​°C at 150 ​rpm. Following incubation, foley catheters sections were transferred to a 24-well cell-culture plate (CytoOne Plate, Starlab, UK) containing 1.5 ​mL AUM [38]. Phage cocktail (1 ​× ​10^2^ ​PFU/mL) was added at the initiation of biofilm formation, where it was diluted 1 in 7 in 1.5 ​mL AUM. Biofilms with and without phage cocktail were incubated statically for 16 ​h at 37 ​°C. After 16 ​h the biofilms were considered mature, as increases in viability had plateaued (Fig. S1). Before antimicrobial treatment, catheter sections were rinsed in PBS to remove any planktonic or loosely adhered cells. Meropenem, mecillinam, or trimethoprim were then added at 164 ​mg/L or 64 ​mg/L in AUM to the biofilm. As trimethoprim was dissolved in Dimethyl sulfoxide (DMSO), as an additional control, DMSO was added to AUM without trimethoprim. Meropenem and mecillinam stocks were dissolved in water. After the addition of drug, biofilms were further incubated for 5 ​h before being removed for analysis, to allow the drugs to penetrate into the biofilm, mirroring the previous microtitre plate experiments. Untreated controls and negative controls were included in every experiment. The experiments were performed in triplicate.

### Biofilm viability analysis

Media and treatments were removed from the biofilm by pipetting off overlying media followed by rinsing in PBS, and viability was assessed using 10 ​μg/mL resazurin sodium salt. Briefly, resazurin salt was diluted in CAMHB from a x100 stock solution. This was then applied to the biofilms, 200 ​μL for cell-culture plate biofilms or 1.5 ​mL for Foley catheter biofilms, and incubated for approximately 2 ​h at 37 ​°C. Absorbance was measured at OD _570 nm_ and _600nm_ and used to calculate the percentage viability, relative to negative controls incubated in each plate [40].

For the Foley Catheter biofilms, the viability was also analysed by incubating the treated section of catheter in 2 ​mL CAMHB for 2 ​h at 37 ​°C at 150 ​rpm. This regrowth bacterial suspension was then used for Miles and Misra counts to assess the CFU/mL. Briefly, the bacterial suspension was serially diluted 1 in 10 and then 10 ​μL plated out on LB agar plates in triplicate. The plates were then incubated overnight at 37 ​°C, the colonies were counted and used to calculate the bacterial concentration in original suspension.

### Statistical analysis

Graphs were produced in MatLab (MatLab R2020a) and GraphPad Prism (Prism version 8.2.4). Unpaired two-tailed t-tests were used to establish significant differences between treated samples and untreated controls. Percentage viability scores were log_2_ transformed before statistical analysis took place. A 2-way ANOVA with Sidak’s multiple comparison test was used to establish any differences between phage infection in different bacterial growth media. Statistical significance was achieved if p ​< ​0.05.

## Results

Single phage treatment is able to prevent biofilm formation, but the phage cocktail has a wider range of activity.

**Fig. 1:**
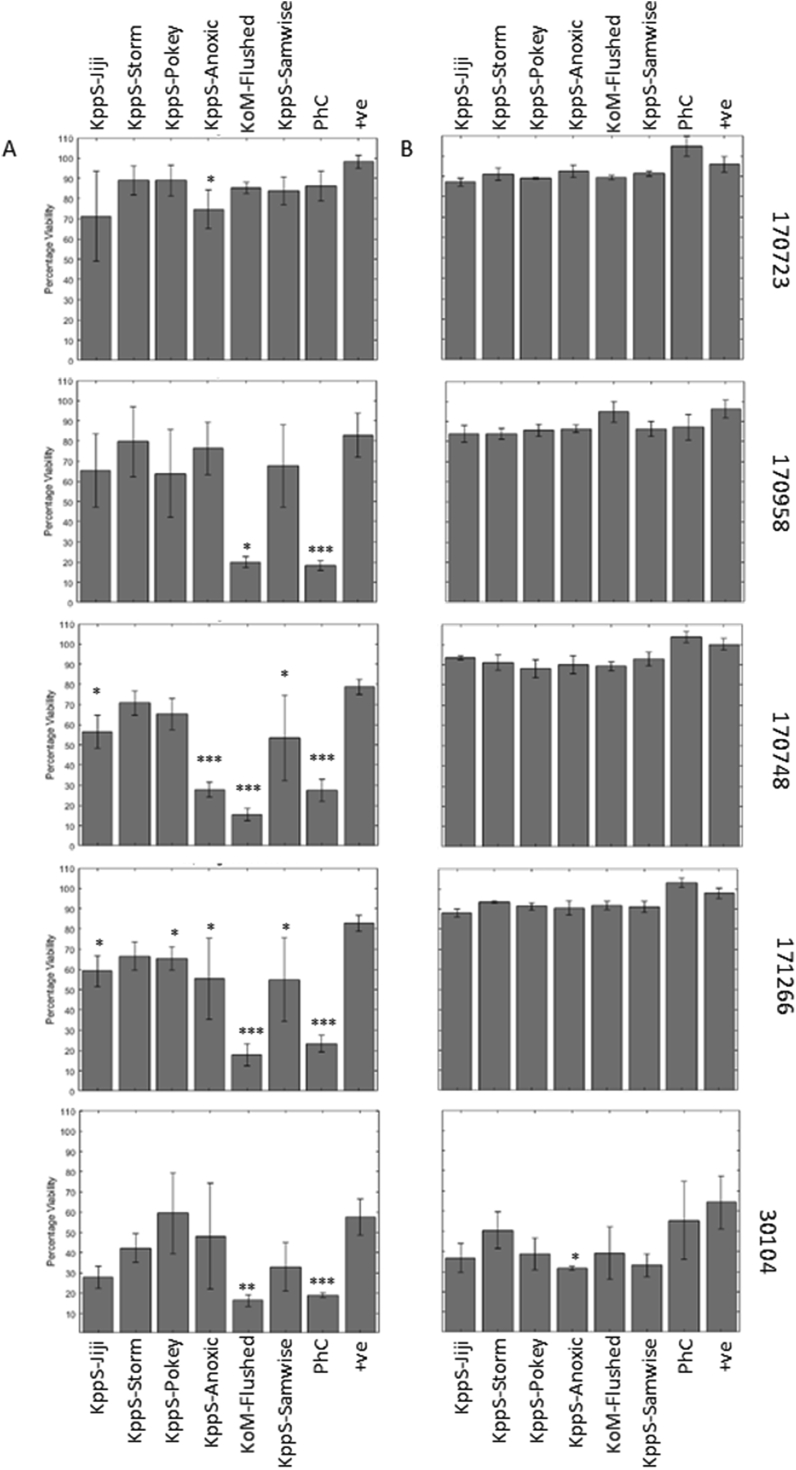
Individual phage and phage cocktail are able to reduce the viability of some, but not all, *Klebsiella* biofilms. Phage were added to biofilms either individually or combined in a cocktail (PhC) at the start (A) of biofilm formation, as a preventative, or for 5 ​h (B) to a mature *Klebsiella* biofilms, as described in the methods. Biofilms were analysed using the resazurin viability stain. Some individual phage were able to reduce biofilm viability when added at the start of biofilm formation (A), and in four strains the cocktail was effective when added at this point. When used on a mature biofilm (B), there was very limited effects on biofilm viability. Symbols denote significant difference (t-test); ∗ compared to positive control, ∗ denotes p value ​≤ ​0.05, ∗∗, p ​≤ ​0.01, ∗∗∗, p ​≤ ​0.001.

Phage had been added to the biofilm grown in CAMHB in 96-well microtitre plates, both at the initiation of biofilm formation (Fig. 1A), and after biofilms had matured (16h, Fig. S1), for a treatment period of 5 ​h (Fig. 1B). This treatment period had been selected in optimisation stages (Fig. S2).

Single phage treatments were able to prevent biofilm formation in a number of strains (Fig. 1A), although strains varied in susceptibility to individual phage. Phage KoM-Flushed had the widest host range, being able to significantly reduce biofilm formation in four out of five strains (170748, 170958, 171266, and 30104), this correlated to a decrease in viability from 60 to 80% with no treatment down to 15–20% biofilm viability with phage treatment, a 40–60% drop. Phage KppS-Anoxic was able to significantly reduce biofilm viability in three strains (170748, 170723, 171266) equal to 20–50% drops, while KppS-Samwise and KppS-Jiji both caused significant reductions in 171266 and 170748, equal to 25–50% drops. Phage KppS-Pokey was only able to significantly reduce biofilm formation by 15% in 171266. Finally, KppS-Storm was unable to cause any significant reductions in biofilm formation in any strains. When these phages were combined to create a phage cocktail, this treatment significantly reduced biofilm formation in all strains except 170723.

When added onto a mature biofilm (Fig. 1B) there were no significant reductions in the viability of the biofilm, with the exception of KppS-Anoxic in strain 30104. Therefore, at this point the results already showed that phage treatment is more effective at preventing biofilm formation rather than penetrating and killing a mature biofilm community. Higher phage PFUs were not tested, but may improve the efficacy of phage treatment against mature biofilms, however we concentrated our efforts on the promising prevention strategy.

Combination of the phage cocktail and meropenem is more effective than monotreatments.

**Fig. 2:**
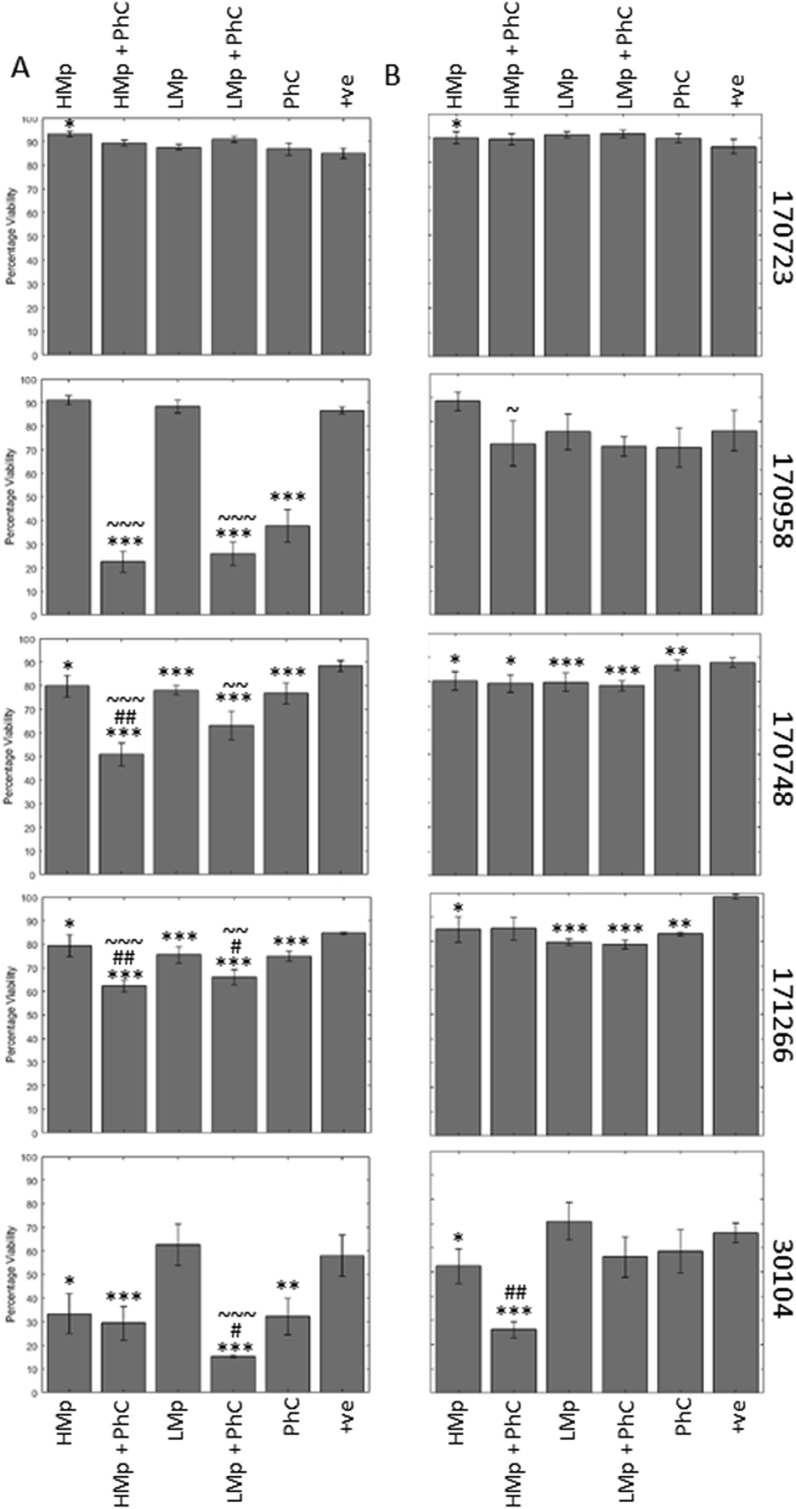
There is synergy between phage cocktail and meropenem when used to prevent biofilm formation. Phage treatments when added at the start (A), as a biofilm prevention strategy, or for 5 ​h (B) to the mature *Klebsiella* biofilms as described in the methods. Biofilms were analysed using the resazurin viability stain. *Klebsiella* strain 170723 had no significant reductions in biofilm viability, however all other strains showed some reductions, and there appeared to be a synergistic effect when used in combination with meropenem. Symbols denote significant difference (t-test); ∗ compared to positive control, # compared to corresponding phage cocktail treatment, and ~ compared to corresponding meropenem treatment. ∗/#/~ denotes p value ​≤ ​0.05, ∗∗/##/~~, p ​≤ ​0.01, ∗∗∗/###/~~~, p ​≤ ​0.001.

Biofilms were again grown in CAMHB in 96-well microtitre plates and challenged with two different doses of meropenem, the phage cocktail, and a combination of meropenem and phage cocktails (Fig. 2). Meropenem concentrations were selected based on average MIC data (Table S2), strains had an increased MIC in biofilm assays compared to planktonic. The phage cocktail was again added at the start of biofilm formation (Fig. 2A) or onto a mature biofilm (Fig. 2B), while meropenem treatments were added after the biofilm had matured. This was intended to replicate two clinical senarios; a phage coated product where phage prevent biofilm formation, and a traditional phage infusion where phage interact with a preformed biofilm. As antibiotics would only be given upon clinical signs of infection, therefore would only be applied to a mature biofilm, these were added after 16 ​h. To assess the combined treatment effects we use the terms defined by Ref. [41]: synergy, facilitation and antagonism. The term synergy denotes a reduction in *Klebsiella* biofilm by the combined treatments that is greater than anticipated based on the sum of the individual treatments. Facilitation describes a reduction in biofilm by the combined treatment that surpasses either individual treatment, but is less than the combined sum of the treatments. Antagonism refers to when the combined treatment effect is worse than the individual treatments. The lytic spectra of the phage is listed in Table S1., but this does not appear to correlate to the antibiofilm activity of the phage cocktail.

As in previous tests, 170723 was unaffected by the phage cocktail, as well as the meropenem treatment at both time points. In fact, we saw antagonism, with a small, yet significant increase in viability when the biofilm was exposed to high levels of meropenem.

When the phage cocktail was added at the start we saw synergy between meropenem and the phage cocktail for the other four strains (170748, 170958, 171266, and 30104). These were not seen in the mature biofilm. No significant reduction compared to the control was seen for 170958 in the mature biofilm. Strains 170748 and 171266 showed individual treatment effects in the mature biofilm, but no additional combined, while strain 30104 showed synergy between the highest level of meropenem and the phage cocktail in the mature biofilm.

The type strain 30104 does not form as robust a biofilm in comparison to the other strains (Fig. S3), as demonstrated by the comparatively lower percentage viability in the positive control compared to the other *Klebsiella* strains. However, the phage cocktail alone was still able to cause a significant reduction. On the mature biofilm, these effects were less evident, and the phage cocktail was not effective.

### Phage infection varies dependent on growth media

Having demonstrated the impact of the phages on biofilm formation in the standard CAMHB-well plate model, we moved forwards with the development of a more reflective *in vitro* model of CAUTI, by investigating phage infection in a series of rich and more minimal growth media. We continued with our use of CAMHB, and additionally tested in LB, M9 supplemented with glycerol as a carbon source, and AUM. We suspected that CAMHB as a rich media would not provide realistic results, but had concerns if phage infection would still occur in more minimal and restrictive growth media.

**Fig. 3:**
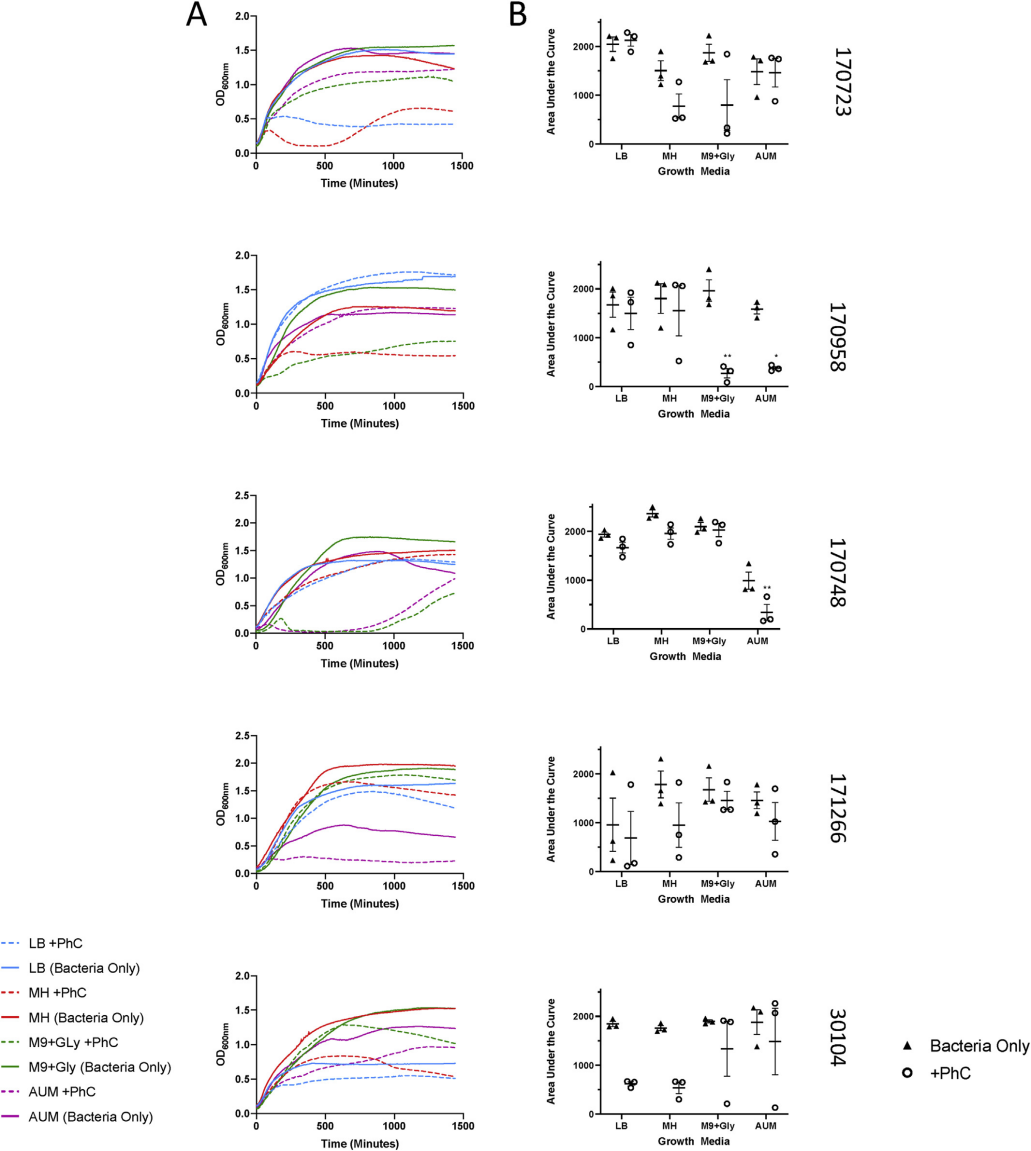
Infection curves and area under the curve analysis for five *Klebsiella* strains infected with the same phage cocktail in four different media. Infection of five *Klebsiella* strains was performed as described in the methods and monitored spectrophotometrically over 24 ​h (A). Bacteria only controls were performed alongside bacteria infected with phage cocktail. Line displays the mean optical density, error bars display standard error across biological triplicates. Area under the curve analysis was performed on the curves (B) to analyse differences in growth rate between media and in presence/absence of phage cocktail. ∗ Denotes significant difference (p ​< ​0.05) between bacterial growth with and without phage cocktail, ∗ denotes p value ​≤ ​0.05, ∗∗, p ​≤ ​0.01, ∗∗∗, p ​≤ ​0.001.

The phage cocktail was used to infect each bacterial strain planktonically in four different growth media, LB, CAMHB, M9 supplemented with 5 ​mM glycerol, and AUM (Fig. 3A). As rates of bacterial growth also differ in each media, the ratio of area under the curve (AUC) in a bacteria only control and bacteria infected with phage was calculated in each media (Fig. 3B).

An ANOVA with Sidak’s multiple comparison tests was used to analyse the data. The data was analysed for interaction effects, and in all but 170958, the two factors (phage and media) did not interact. Therefore, in the four remaining strains, the effects of phage and media were independent of each other.

The media accounted for variance in strains 170723 (p value ​= ​0.0185, 32.95%), 170748 (p value ​< ​0.0001, 83.51% variance) and was near significant for 170958 (p value ​= ​0.0542, 17.23% variance). The presence or absence of phage accounted for variance in the type strain 30104 (p value ​= ​0.0021, 37.12%), 170723 (p value ​= ​0.0390, 12.44%), 170748 (p value ​= ​0.0008, 7.145%) and 170958 (p value ​= ​0.0006, 33.5% variance). The results in 170958 were difficult to interpret as there was interaction between the two factors (p value ​= ​0.0349, 20.07% variance).

For 30104 there were no significant differences between±phage in each media, but it can be seen that there was a more marked decrease in AUC in the LB and MH media. Similarly but in different media for 170723 there was an observable reduction of the AUC in MH and M9/Gly. For 170748 there were modest decreases in AUC with phage in media LB and MH, but only the decrease in AUM media was significant (0.0059). In strain 171266, neither the effects of media or phage were considered significant. However, in each media there is a slight decrease in the mean AUC in the presence of phage. There was also a significant decrease in 170958 AUC with phage in both M9/Gly (0.0019) and AUM (0.0262).

Overall, these results show that the nutritional environment that the host bacteria are growing in can influence the cell lysis by phage, although this varies dependent on the host strain. Therefore, we decided to continue our experiments using AUM for a growth media, as this both allowed phage infection to occur in a number of strains planktonically, as well as being close to the clinical situation we are attempting to mimic.

### Biofilms in the *in vitro* foley catheter model

**Fig. 4:**
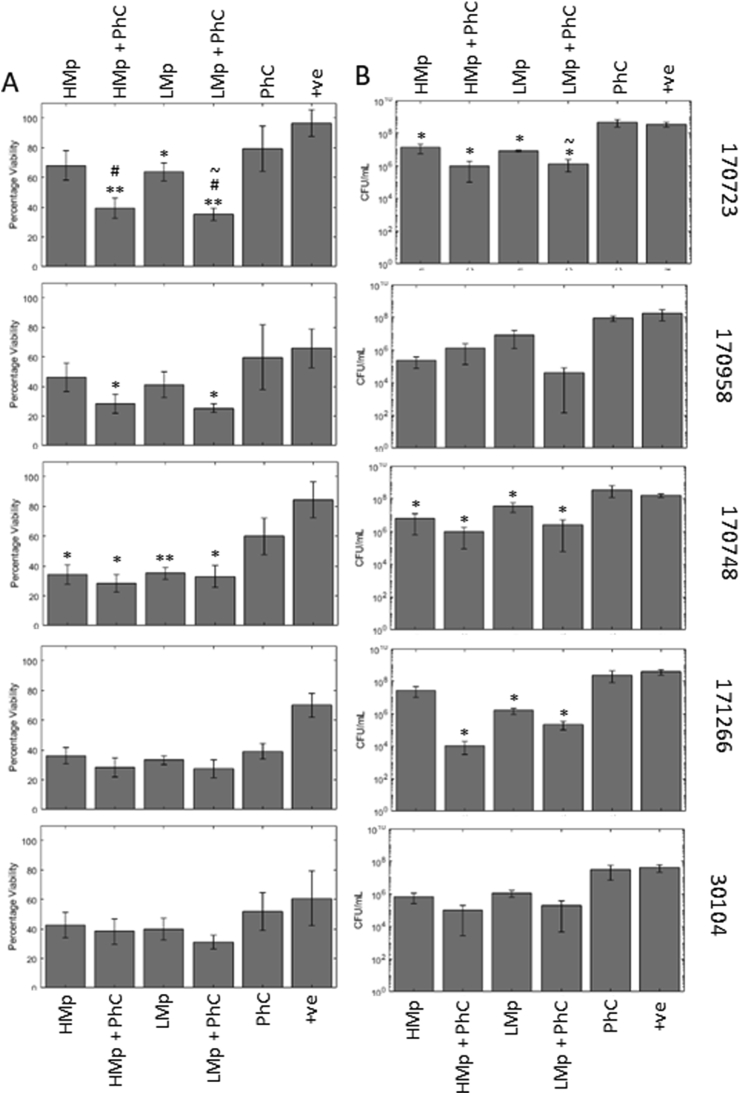
In a more complex model (Foley catheter *in vitro* model) treatments are less effective but are still able to cause some reduction in biofilm viability and sloughing. Phage treatment was combined with meropenem in a Foley catheter *in vitro* model. Biofilm reduction was measured by viability assay (A) and CFU counts (B). Treatments were able to reduce viability and sloughing, most notably in strain 170723 which had previously not responded to the same treatment in previous testing. Symbols denote significant difference (t-test); ∗ compared to positive control, # compared to corresponding phage cocktail treatment, and ~ compared to corresponding meropenem treatment. ∗/#/~ denotes p value ​≤ ​0.05, ∗∗/##/~~, p ​≤ ​0.01, ∗∗∗/###/~~~, p ​≤ ​0.001.

The meropenem treatment in combination with the phage cocktail significantly reduced the viability of the biofilm in three clinical strains; 170723, 170958, and 170748 (Fig. 4A). For 170723, the combined effect was synergistic, while facilitation was observed in strains 170958, and 170748. For strains 171266 and 30104, the reductions in biofilm viability in response to all treatments were not significant (Fig. 4A).

The second analysis for meropenem was of colony forming units counts of bacterial cells sloughed off from the biofilm (Fig. 4B). This is linked to what is within the biofilm itself, as well as giving an indication to likelihood of infection to disperse from the biofilm and disseminate after treatment. However, it is unclear if these sloughed off cells should be considered as planktonic cells or a small piece of biofilm, this is an important consideration because phages infect planktonic cells differently to intact biofilms. Correspondingly the results are less clear, with only 170723, 170748, and 171266 showing significant reductions in colony counts after either meropenem or combination treatments. Positive controls contained 3.73 ​× ​10^8^, 1.79 ​× ​10^8^, 1.63 ​× ​10^8^, and 3.86 ​× ​10^8^, 4.38 ​× ​10^7^ ​CFU/mL for 170723, 170958 170748, 171266, and 30104 respectively. Combination treatments caused an average 2.69 log reduction (range 1.81–4.16 logs) while phage alone caused a 0.05 increase (range −0.36-0.28) and drug alone caused a 1.59 log reduction (high 1.74, 1.14–2.88, low, 1.45, 0.65–2.01). Only 170723 treated with low level meropenem and the phage cocktail (1.37 ​× ​10^6^ ​CFU/mL) showed a significant reduction in colony forming units counts compared to low level meropenem treatment alone (8.11 ​× ​10^6^ ​CFU/mL).

**Fig. 5:**
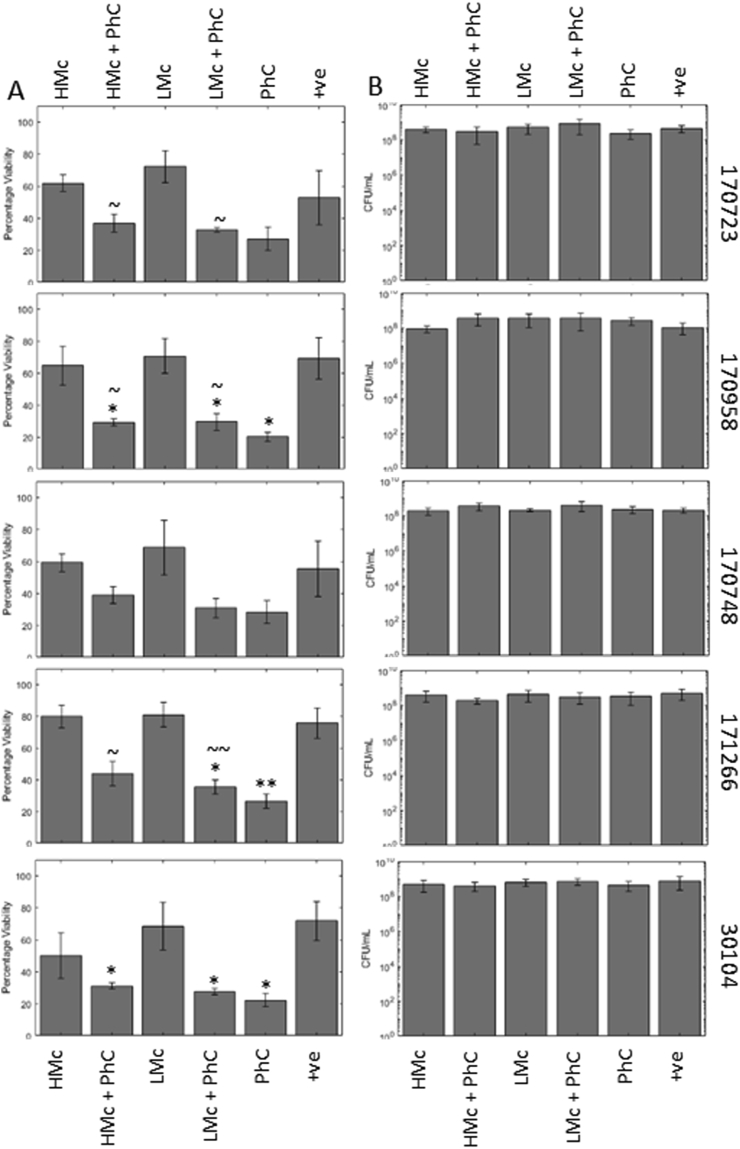
Mecillinam was not effective at reducing *Klebsiella* biofilms by either measure, and phage cocktail was more effective alone. Phage treatments were combined with mecillinam, a second line drug used for treatment of CAUTI in the *in vitro* Foley catheter model. The treatment effect was measured by viability assay (A) and CFU counts (B) as described in the methods. The viability staining showed that phage cocktail alone was more effective compared to drug alone or in combination. All effects were lost when measured by CFU counts. Symbols denote significant difference (t-test); ∗ compared to positive control, # compared to corresponding phage cocktail treatment, and ~ compared to corresponding mecillinam treatment. ∗/#/~ denotes p value ​≤ ​0.05, ∗∗/##/~~, p ​≤ ​0.01, ∗∗∗/###/~~~, p ​≤ ​0.001.

The next drug used was mecillinam, a second-line antibiotic used for CAUTI [42]. *Klebsiella* should be intrinsically resistant to this penicillin antibiotic, therefore it is not surprising that the phage treatment was driving the reduction in viability of the biofilm here (Fig. 5A). Phage treatment repeatedly resulted in the lowest *Klebsiella* viability significantly lower than the control for 170958, 171266 and 30104, but were not significantly different from the combined mecillinam and phage treatment. In strains 170723, 170958, and 171266, the combined mecillinam and phage treatment resulted in were significantly lower biofilm viability compared to the mecillinam alone. The colony forming unit analysis showed no significant differences regardless of treatment (Fig. 5B).

Trimethoprim was the final drug used, a first-line antibiotics in the treatment of CAUTI [42]. As the drug needed to be suspended in DMSO, all other treatments and the positive control also had DMSO added to control for any additional effects.

**Fig. 6:**
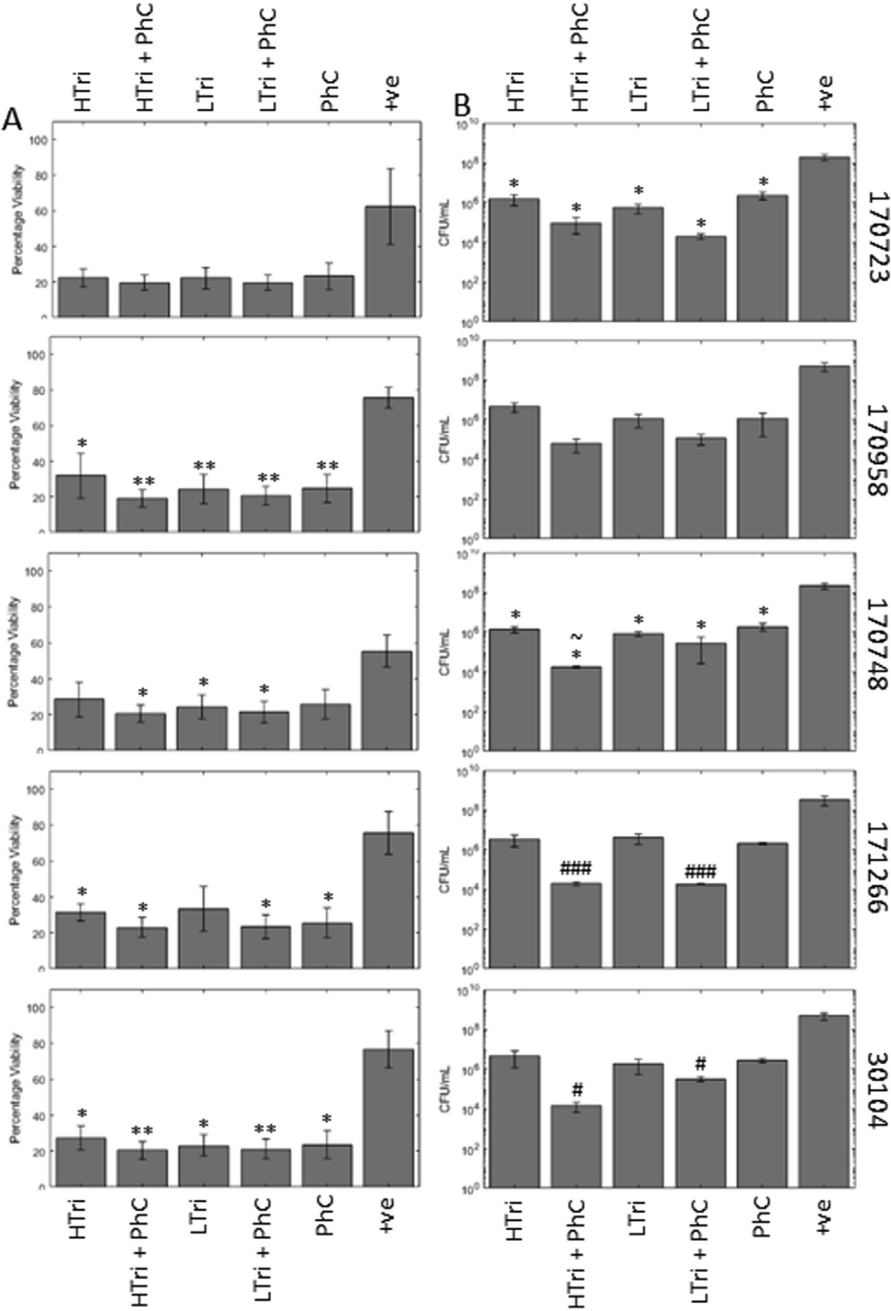
Trimethoprim is effective at reducing *Klebsiella* biofilms and CFU counts show facilitation with phage cocktail. Phage treatments combined with trimethoprim, a first line drug for CAUTI, measured by viability assay (A) and CFU counts (B) in an *in vitro* Foley catheter model. In the viability assay, all treatments were approaching the lower limit of detection by the assay, therefore it is difficult to see any facilitation or synergy with the phage treatment. However, this is more evident in the CFU counts where reductions are greater in the combination treatments. Symbols denote significant difference (t-test); ∗ compared to positive control, # compared to corresponding phage cocktail treatment, and ~ compared to corresponding trimethoprim treatment. ∗/#/~ denotes p value ​≤ ​0.05, ∗∗/##/~~, p ​≤ ​0.01, ∗∗∗/###/~~~, p ​≤ ​0.001.

In strain 170958 and 30104, all treatments significantly decreased the percentage viability of the biofilms. In 170748, high trimethoprim with the phage cocktail, low trimethoprim, and low trimethoprim with phage significantly reduced the viability of the biofilm (Fig. 6A). In strain 171266, all treatments except low trimethoprim were able to significantly reduce the viability of the biofilm. In strain 170723, despite reductions in viability, none of the changes caused by the treatments were statistically significant. However, when analysed by CFU counts, this change became significant (Fig. 6B), with 2.10 and 2.57 log reductions in the high and low trimethoprim, and 3.31 and 4.01 log reductions when they were combined with PhC. Strain 170748 also had significantly lower CFU counts with every treatment, with the addition of high trimethoprim and the phage cocktail (1.70 ​× ​10^4^ ​CFU/mL) being a more significant reduction compared to trimethoprim alone (1.42 ​× ​10^6^ ​CFU/mL). In strains 171266 and 30104, the combined treatments significantly reduced the CFU counts (171266, 4.21 and 4.24 log reductions for high and low trimethoprim with PhC, 30104, 4.54 and 3.18 log reductions) compared to the phage treatment alone (171266, 2.18 log reduction, 30104, 2.42, log reduction). Strain 170958 had no significant changes to the CFU counts.

## Discussion

In this paper we have shown that antibiotics can be used in combination with preventative phage cocktail treatments, synergistically to reduce the amount of biofilm formation on urinary tract catheters. This shows the potential of using phages to prevent *Klebsiella* CAUTI and the further complications associated with *Klebsiella* infections.

We have also shown that using appropriate models is important, particularly in respect to mimicking *in vivo* conditions. This has been shown to be important for antibiotic treatments, but even more so for phage infection, evidenced by the initial experiments comparing media for planktonic phage infection and the varying response of strain 170723 in the two main biofilm models. The changes in phage efficacy may be linked to corresponding changes in the availability of phage binding sites such as the capsules of *Klebsiella*, it has previously been demonstrated that encapsulation can be inhibited by carbohydrate restriction and the ionic strength of growth media [43,44]. Although the model presented here remains relatively simple, we have already shown that increasing complexity is important in testing new treatments. We theorise that on exposure to different media and surfaces, *Klebsiella* may vary their capsule composition and cell surface receptors in the different environments and nutrient availability [[45], [46], [47], [48]].

Mixtures of phages, commonly known as phage cocktails, are used for therapeutic reasons over a lone phage for the same reason that drug cocktails are often used in particularly recalcitrant infections such as HIV or TB. Phage cocktails have a broader host range than individual phages, reducing the chance of phage resistance emerging, which can maximise the efficiency of therapy [49]. Phage resistance is a common phenomenon in the natural predator-prey relationship between phages and bacteria. More specifically in the case of *Klebsiella* biofilms, phages may possess depolymerase enzymes [50,51] that are able to degrade either the bacterial capsule or the extracellular matrix that protects cells within the biofilm. Depolymerase action can then facilitate the penetration of antimicrobial drugs into the biofilm or may uncover receptors for a secondary phage infection [52]. In this way, phage cocktails are protected from resistance developing and the likelihood of synergistic interactions increases. A phage cocktail was composed of six phages that had been previously characterised by our group [36]. The phages selected represented the broadest host range phages and most genetically diverse of the characterised phages in the laboratory at the time the experiments were conducted. In this study, we shown that in a number of cases, phages combined with an antimicrobial drug were able to cause a significant reduction in biofilm viability compared to each treatment alone displaying either synergy or facilitation. Our results indicate that the treatments have a synergistic action when used together, but the precise mechanism behind is yet to be elucidated. To further characterise this synergistic effect, investigations into the pharmacokinetics of the treatments would need to be established to improve the quantification and nature of the observed effects of combining phage and antimicrobials.

Our study focusses on the use of meropenem, a carbapenem antibiotic, selected based on MIC data. In addition, we have used trimethoprim, a sulphonamide, and mecillinam, a penicillin, which are both recommended in the NICE guidelines for use in treatment of CAUTI [42]. Trimethoprim was particularly effective against the *Klebsiella* strains used in this study. So much so, that in the viability assay the results were approaching the lower limit of detection of the assay, making it difficult to detect any additional benefit of co-treatment with phage. However, these effects were better seen in the colony forming units counts of cells sloughed off the biofilm, highlighting the benefits of using multiple methods to measure treatment effects. Conversely, mecillinam was not at all effective against the strains. This is not surprisingly as *Klebsiella* have long been recognised as intrinsically resistant to penicillin antibiotics [53]. The hypothesis behind choosing mecillinam was that there may be some synergistic activity between the phage and drug which would overcome this resistance. This has been previously demonstrated where a phage’s depolymerase enzymes are able to degrade the matrix and capsule of a bacteria, allowing antibiotic penetration where it would not normally occur [54,55]. Unfortunately, this was not the case here, despite the fact that phage KppS-Pokey has been predicted to encode a depolymerase [36]. It is well characterised that sub-inhibitory concentrations of antibiotics can stimulate biofilm formation [56]. Instead this appears to be the effect seen here, where the phage cocktail is the driving force in the reduction of viability and the presence of mecillinam only stimulates the biofilm [14,15]. While the use of a phage cocktail can theoretically assist drug penetration and increase efficacy, it cannot substitute selection of an appropriate antibiotic.

In the development of our *in vitro* CAUTI model, we investigated phage infection in different nutritional environments. The effects of nutritional environment were briefly investigated by Storms et al. in the development of the virulence index, a method of quantifying phage virulence relative to the natural host growth rate [39]. In the study of [39] it was also shown that temperature had an effect on phage virulence alongside growth media, as well as the same phage having different dynamics in secondary host strains. These are all factors that need to be taken into consideration when optimising phage cocktails for use outside of the lab. In this work, all experiments were carried out at 37 ​°C to mimic the temperature experienced in human infections.

There have been a number of studies for other urinary tract pathogens, where phages have been shown to be successful prevention strategies [28,29,57,58], sometimes in combination with a benign biofilm [26]. These results have been shown in clinical or urinary tract pathogens (but not *Klebsiella*) without any negative side effects being found to be associated with the phage treatment [30]. Although larger studies are required before phage treatments can overtake antibiotics, the current evidence shows phage treatments are accepted by patients and can show efficacy [30,59,60]. A review of current phage therapeutics, including urinary tract catheters can be found here [60]. We propose that similar phage therapeutics for urinary tract catheters could be developed for *Klebsiella*. Phages have already been shown to be a valid method of eradicating *Klebsiella* biofilms, even in multi-resistant strains [[61], [62], [63]], depolymerases isolated from phage have also shown to have efficacy [64]. Our *Klebsiella* model shows that phage cocktails have promise in the prevention of *Klebsiella* CAUTIs, which would prevent complications in vulnerable patients and prolong the time urinary catheters can remain *in situ*.

## Conclusions

This is the first time our *in vitro* CAUTI model has been presented, and the first time the efficacy of antibiotics in combination with phage cocktail has been tested on *Klebsiella* CAUTI biofilms. Our model provides a simple, cheap *in vitro* model that is more realistic and reflective for determining phage efficacy than 96 well plate models in standard rich media. While it does lack certain properties, such as sheer stress from urine passing over the biofilm, it provides the correct nutritional environment and attachment surface for CAUTI biofilm growth. We believe it provides a solid starting block to test promising phage, and other antimicrobial, CAUTI therapies Phage cocktails shows synergy with the current CAUTI antimicrobial treatments, trimethoprim and meropenem, to help prevent biofilm formation by *Klebsiella* species in catheters. This has important implications for clinical therapy, to offer an alternative treatment for antimicrobial resistance *Klebsiella*.

## CRediT authorship contribution statement

**Eleanor M. Townsend:** Conceptualization, Methodology, Investigation, Writing - original draft, Visualization. **John Moat:** Methodology, Resources, Supervision. **Eleanor Jameson:** Conceptualization, Writing - review & editing, Visualization, Supervision, Funding acquisition.

## Declaration of competing interest

The authors declare that they have no known competing financial interests or personal relationships that could have appeared to influence the work reported in this paper.
