# Supplementary material for: CAUTI’s next top model – Model dependent Klebsiella biofilm inhibition by bacteriophages and antimicrobials
Source: Biofilm. 2020 Nov 11;2:100038. doi: 10.1016/j.bioflm.2020.100038 (PMC7762788; doi:10.1016/j.bioflm.2020.100038)
Supplement: Multimedia component 1 [file mmc1.docx]

| ***Klebsiella* strain.** | | **Spot testing** | | | | | | | **Planktonic susceptibility to PhC** |
| --- | --- | --- | --- | --- | --- | --- | --- | --- | --- |
| **Strain number** | **Capsule type** | **KppS-Samwise** | **KppS-Jiji** | **KppS-Storm** | **KppS-Pokey** | **KppS-Anoxic** | **KoM-Flushed** | **PhC** |  |
| 30104 | KL3 | Y | Y | Y | Y | Y | Y | Y | Y |
| 170723 | KL2 | N | Y | N | N | Y | Y | N | N |
| 170958 | KL28 | N | N | N | N | N | Y | Y | Y |
| 170748 | O1v1 | Y | Y | Y | N | Y | N | N | N |
| 171266 | OL104 | Y | Y | Y | N | Y | N | N | N |
