# Supplementary material for: CAUTI’s next top model – Model dependent Klebsiella biofilm inhibition by bacteriophages and antimicrobials
Source: Biofilm. 2020 Nov 11;2:100038. doi: 10.1016/j.bioflm.2020.100038 (PMC7762788; doi:10.1016/j.bioflm.2020.100038)
Supplement: Multimedia component 2 [file mmc2.docx]

| ***Klebsiella* strain.** | **Meropenem (mg/L)** | | |
| --- | --- | --- | --- |
|  | **Planktonic MIC** | **MBIC_50_** | **MBIC_90_** |
| 30104 | 0.25 | 4096+ | 4096+ |
| 170723 | 0.125 | 128 | 4096+ |
| 170958 | 1 | 4096+ | 4096+ |
| 170748 | 1 | 64 | 4096+ |
| 171266 | 0.5 | 64 | 4096+ |

Reference

[66] Gordon %J Antimicrobial agents Ramage, and chemotherapy Townsend Eleanor M., SherryLeighann, Ryan Kean, Hansom Donald, Mackay William G., Williams Craig, et al.’Implications of antimicrobial combinations in complex wound biofilms containing fungi. AAC; 2017. 00672-17.
