# Supplementary material for: CAUTI’s next top model – Model dependent Klebsiella biofilm inhibition by bacteriophages and antimicrobials
Source: Biofilm. 2020 Nov 11;2:100038. doi: 10.1016/j.bioflm.2020.100038 (PMC7762788; doi:10.1016/j.bioflm.2020.100038)
Supplement: Multimedia component 5 [file mmc5.docx]

[67]Townsend Eleanor M., Sherry Leighann, Rajendran Ranjith, Hansom Donald, Butcher John, Mackay William G., et al. ’Development and characterisation of a novel three-dimensional inter-kingdom wound biofilm model 2016;32:1259–1270.
