# Supplementary material for: CAUTI’s next top model – Model dependent Klebsiella biofilm inhibition by bacteriophages and antimicrobials
Source: Biofilm. 2020 Nov 11;2:100038. doi: 10.1016/j.bioflm.2020.100038 (PMC7762788; doi:10.1016/j.bioflm.2020.100038)
Supplement: Supplementary file 4 [file mmc3.docx]

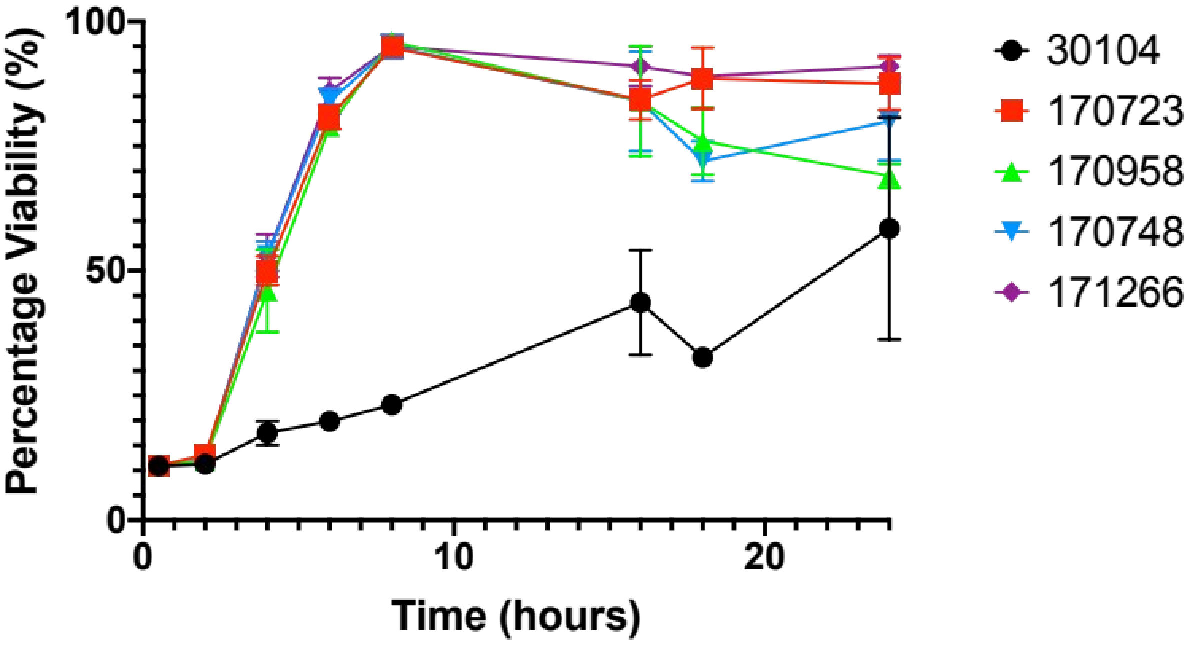
Percentage Viability (%)
100
50
0
0
10
20
Time (hours)
30104
170723
170958
170748
171266
