# Supplementary material for: CAUTI’s next top model – Model dependent Klebsiella biofilm inhibition by bacteriophages and antimicrobials
Source: Biofilm. 2020 Nov 11;2:100038. doi: 10.1016/j.bioflm.2020.100038 (PMC7762788; doi:10.1016/j.bioflm.2020.100038)
Supplement: Supplementary file 5 [file mmc4.docx]

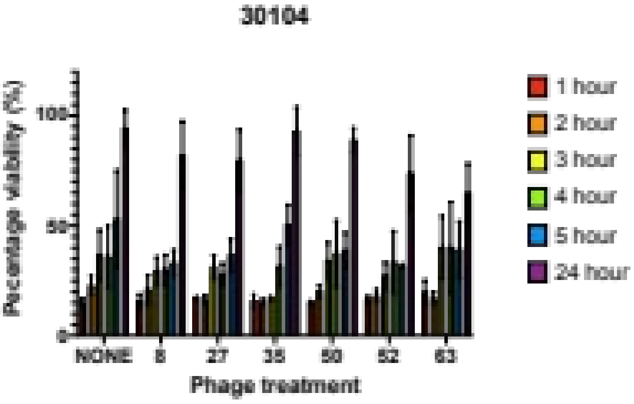
30104
Pecentage viability (%)
0
50
100
NONE
8
27
35
50
52
63
Phage treatment
1 hour
2 hour
3 hour
4 hour
5 hour
24 hour

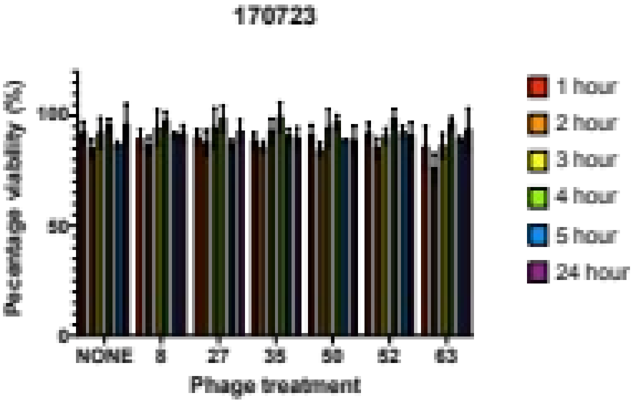
170723
Pecentage viability (%)
0
50
100
NONE
8
27
35
50
52
63
Phage treatment
1 hour
2 hour
3 hour
4 hour
5 hour
24 hour

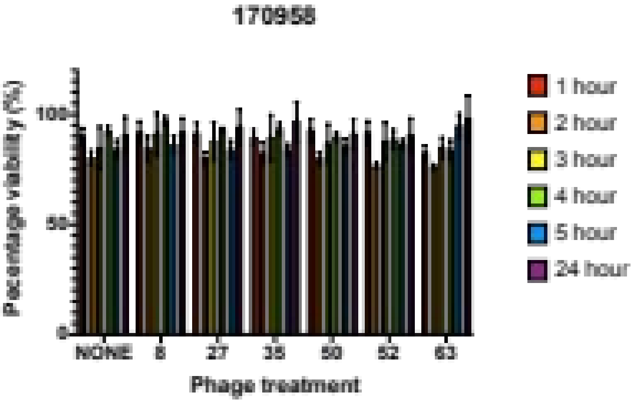
170958
Pecentage viability (%)
0
50
100
NONE
8
27
35
50
52
63
Phage treatment
1 hour
2 hour
3 hour
4 hour
5 hour
24 hour

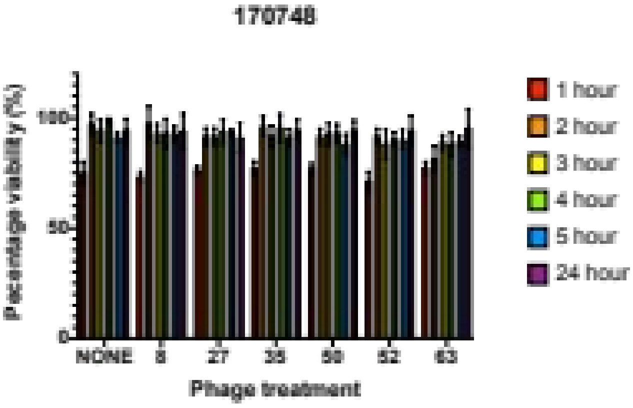
170748
Pecentage viability (%)
0
50
100
NONE
8
27
35
50
52
63
Phage treatment
1 hour
2 hour
3 hour
4 hour
5 hour
24 hour

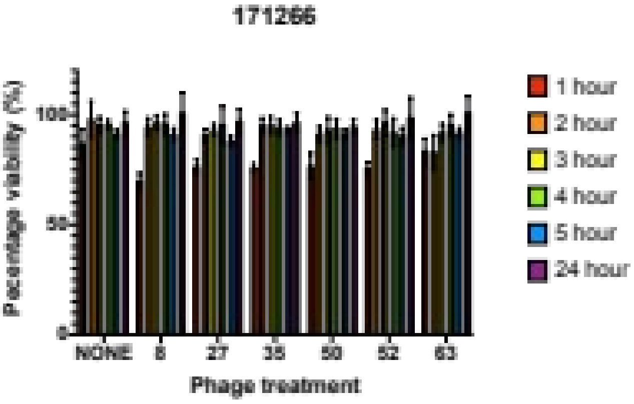
171266
Pecentage viability (%)
0
50
100
NONE
8
27
35
50
52
63
Phage treatment
1 hour
2 hour
3 hour
4 hour
5 hour
24 hour
